# Dealing With Brain MRI Findings in Pediatric Patients With Endocrinological Conditions: Less Is More?

**DOI:** 10.3389/fendo.2021.780763

**Published:** 2022-01-12

**Authors:** Francesco Baldo, Maura Marin, Flora Maria Murru, Egidio Barbi, Gianluca Tornese

**Affiliations:** ^1^ University of Trieste, Trieste, Italy; ^2^ Institute for Maternal and Child Health IRCCS “Burlo Garofolo”, Trieste, Italy

**Keywords:** incidentaloma, growth hormone deficiency, central precocious puberty, incidental radiological finding, follow-up

## Abstract

Neuroimaging is a key tool in the diagnostic process of various clinical conditions, especially in pediatric endocrinology. Thanks to continuous and remarkable technological developments, magnetic resonance imaging can precisely characterize numerous structural brain anomalies, including the pituitary gland and hypothalamus. Sometimes the use of radiological exams might become excessive and even disproportionate to the patients’ medical needs, especially regarding the incidental findings, the so-called “incidentalomas”. This unclarity is due to the absence of well-defined pediatric guidelines for managing and following these radiological findings. We review and summarize some indications on how to, and even if to, monitor these anomalies over time to avoid unnecessary, expensive, and time-consuming investigations and to encourage a more appropriate follow-up of brain MRI anomalies in the pediatric population with endocrinological conditions.

## Introduction

The widespread use of brain magnetic resonance imaging (MRI) has put at our disposal incredibly accurate images that give us the chance to improve our diagnostic competence. However, this widespread use of neuroimaging has led to a remarkable increase of incidental radiological findings, the so-called “incidentalomas”, which may generate interpretation uncertainty for radiologists and clinicians (1, 2). Here we describe two clinical cases to exemplify this context.

## Clinical Scenario 1

A boy was referred for short stature at the age of 5 years. His height was -2.3 standard deviation score (SDS), his growth rate was -2.6 SDS, and his bone age was 3 years. His previous medical history was unremarkable. Two stimulation tests were performed with arginine and insulin, and peak values of growth hormone (GH) were pathological (<8 ng/mL) in both, supporting the diagnosis of GH deficiency (GHD). No other pituitary deficits were found on laboratory tests. A brain MRI under sedation was performed before starting treatment, highlighting the presence of “pituitary stalk interruption syndrome”. An annual MRI follow-up was suggested, and the patient underwent a brain MRI every year for 7 consecutive years, through which the radiological finding remained stable.

## Clinical Scenario 2

A girl was referred for precocious puberty at the age of 7 years. On physical examination, Tanner stages B2, Ph2, and A2-3 were observed. Her growth rate was +6 SDS, and her bone age was 9 years. A pelvic ultrasound showed pubertal changes in the uterus and ovaries, and a GnRH stimulation test showed a pathological level of LH peak (> 5 ng/mL) and an LH/FSH ratio >1, confirming the diagnosis of central precocious puberty (CPP). A brain MRI showed the presence of an arachnoid cyst in the parietal lobe, with dimensions of 2 x 3 cm, not compressing nor close to the ventricular system. An annual MRI follow-up was suggested. The patient underwent a brain scan for 4 consecutive years, through which the radiological finding did not show any variation.

## Discussion

Brain MRI is mandatory in many pediatric endocrinological conditions to detect anatomic anomalies and rule out neoplastic lesions (3, 4).

In two of the most frequent conditions in pediatric endocrinology, GHD (prevalence 1:4.000-1:10.000) and CPP (prevalence 1:5.000-1:10.000) (5), brain MRI is requested once stimulation tests confirm the clinical diagnosis (3, 4). Nevertheless, other rare endocrine conditions (such as Cushing syndrome, hyperprolactinemia, gigantism, central hypo- and hyperthyroidism, and diabetes insipidus) unquestionably require a brain MRI in their diagnostic work-up.

In GHD, brain MRI may show characteristic anatomic pituitary abnormalities that can explain the endocrine disorder (such as anterior pituitary dysplasia, pituitary stalk interruption syndrome [PSIS], Rathke cleft cyst [RCC], empty sella, etc.), but can also detect possible tumoral lesions (such as craniopharyngioma or Langerhans cell histiocytosis) (6).

In CPP, the international guidelines state that neuroimaging is mandatory in all males and in females under six years of age since both these groups have a higher chance of presenting brain lesions causing early pubertal development (such as hamartoma, astrocytoma/glioma, germinoma) (7–9). Conversely, MRI is still controversial in females aged 6-8 years: the incidence of cranial abnormalities in this age group is minimal (10–12), even if a possible late cancer diagnosis cannot be excluded by clinical and biochemical parameters only (13).

In both GHD and CPP, identifying anomalies and malformations during brain MRI, including “incidentalomas”, is not infrequent (9, 14). Starting from the clinical scenarios we described, we realized that unrelated brain anomalies, incidentalomas, and anatomic anomalies represent a real problem in the pediatric population. Thus, we asked ourselves whether it was necessary to routinely monitor all these findings after their identification or not, based on the current medical literature. Avoiding unnecessary tests on pediatric patients is not just a matter of cost, both for the health system and the patients’ families, but also of physical and psychological stress for the patients and their families. The image acquisition process for brain MRI lasts from 20 to 30 minutes, and procedural sedation is usually needed in children younger than 7 years of age. Furthermore, unnecessary investigations and tests may postpone the medical evaluation of other patients in real need of consult.

We performed a narrative review of the literature, which was limited to the pediatric population, to identify the most common brain abnormalities in patients with endocrinological conditions who require an MRI scan. We then conducted subsequent research to determine the current management for every specific MRI abnormal finding. In doing so, we decided to include exclusively papers that described cohorts of patients, excluding single case reports or international guidelines on the matter. For rare conditions, if more than one cohort study was identified, we reported the ones with the largest population. The selection of papers was limited to the English language. The following three electronic databases were searched: Pubmed, Scielo, and Scopus. As search string, we used a combination of the condition’s name plus pediatric/children plus management/guidelines/follow up or, alternatively, the condition’s name plus pediatric/children plus MRI/magnetic resonance. We also searched for pediatric guidelines referring to the overall topic of brain incidentalomas *via* the database above reported.

Remarkably, despite the increasing relevance of the matter, we found out that there was a general lack of guidelines on incidentalomas and MRI brain abnormalities in the pediatric population. While the medical literature has vastly discussed this topic in the adult population, only a few studies currently support pediatricians’ decisions on managing these conditions. For example, a 2011 paper by the Endocrine Society formulated some practical guidelines for managing pituitary incidentalomas in adults but clearly stated that they do not apply to the pediatric population (15). On the other hand, most papers on the pediatric population either describe small sample populations or analyze only a single subtype of incidentaloma without considering the remaining anomalies, missing the big picture. The works by Souteiro et al. and Shareef et al. represent the two most extensive studies (2, 16). The first one identified 41 incidentalomas in a cohort of pediatric patients who underwent brain imaging for various reasons, primarily headache. Pituitary hypertrophy was the most common finding (29.3%), followed by arachnoid cysts (17.1%), pituitary adenomas (14.6%), and RCC (12.2%). Remarkably, 56.1% of these patients underwent a radiological reevaluation, but none of them presented dimensional progression (2). The second one described 31 incidental lesions, among which RCC was the most frequent (67.7%), followed by cystic pituitary lesions (19.4%) and microadenomas (12.9%). Only 5 patients had a radiological reevaluation, and lesion growth was never documented (16).

According to the available evidence, we divided the radiological findings into two main groups:

a) **Findings with definite or possible clinical or anatomical relevance**, i.e., conditions involving the sellar region that might affect the endocrine system function. These include:

- *Adenohypophysis hypoplasia*
- *Pituitary stalk interruption syndrome (PSIS)*
- *Ectopic neurohypophysis*
- *Empty sella, complete or partial*
- *Rathke cleft cysts (RCC)*
- *Pituitary adenomas*
- *Craniopharyngiomas*

Overall, these findings have been identified more frequently in GHD than CPP (3, 17). *Arnold Chiari type I malformation*, i.e., the descent of the cerebellar tonsils through the foramen magnum, cannot be included in this group since it does not affect the sellar region. Still, it often has clinical relevance for the patient (18, 19).

Only three findings can potentially grow over time in this category: RCC, pituitary adenomas, and craniopharyngiomas (20–22). There is evidence that craniopharyngioma may progress from a longstanding RCC *via* a transitional stage of extensive squamous metaplasia (23).

The remaining lesions cannot progress and thus do not need radiological follow-up. For example, in the first clinical scenario, no further MRIs should have been performed in a patient with PSIS.

b) **Findings without any relevance that lack a correlation with the endocrinological condition under study**. This group contains the so-called “incidentalomas”, such as:

- *Arachnoid cysts*
- *Epiphyseal cysts*
- *Choroid plexus cysts*
- *Vascular anomalies*
- *Increased hypophyseal volume*

Various studies have followed these findings throughout the years with subsequent MRI (6). However, due to the lack of standardized protocols on how to, and even if to, monitor them, and to a general misunderstanding on their ability to evolve or not, it is not infrequent that these lesions can be troublesome to deal with, leading to a relevant number of pointless tests.

Among incidentalomas, only arachnoid cysts may be worth further radiological evaluations since they can grow to a large size if the retention of cerebrospinal fluid progresses through time. However, a radiological follow-up is recommended only in the presence of a large cyst or if the lesion is located close to the ventricular system, posing a risk of hydrocephalus (24). Therefore, in the second clinical scenario we described, no other exams were needed.

We propose a summary table to manage the most frequent brain anomalies found in pediatric patients affected by GHD and CPP that is now in use in our Institute (**Table 1**) (2, 18, 24–59). This table is provided only as a guide and does not give absolute indications.

**Table 1:** Suggested management of the most frequent brain anomalies found in pediatric patients affected by growth hormone deficiency (GHD) and central precocious puberty (CPP).

MRI Finding	Management and follow-up	Reference
***Findings with definite or possible clinical or anatomical relevance***		
Adenohypophysis hypoplasia	Baseline laboratory tests Not require follow-up imaging studies	(25, 26)
Pituitary stalk interruption syndrome (PSIS)	Baseline laboratory tests Not require follow-up imaging studies	(27, 28)
Ectopic neurohypophysis	Baseline laboratory tests Not require follow-up imaging studies	(29, 30)
Empty sella, complete or partial	Baseline laboratory tests Repeat laboratory tests after 24-36 months (in the absence of clinical manifestations)	(31, 32)
Rathke cleft cyst (RCC)	→ *Symptomatic:* MRI every year for 5 years after the surgical removal of the cyst + Laboratory tests → *Asymptomatic (when >5 mm):* MRI at 1, 3, 5 years + Laboratory tests	(33–35)
Pituitary adenoma	→ *Symptomatic:* Surgical or medical therapy + Follow-up (laboratory test, MRI, visual field evaluation) according to the oncological guidelines → *Asymptomatic:* - Macro (≥10 mm): laboratory tests + visual field evaluation + MRI at 6 months and then every year - Micro (≥5 mm): laboratory tests + MRI every year for the first 3 years and every 1-2 years - Micro (<5 mm): MRI not required	(36–38)
Craniopharyngiomas	Follow-up after surgery ± radio/chemotherapy according to the oncological guidelines	(39–42)
Arnold-Chiari type I	Neurosurgical consultation	(18, 43–45)
***Incidentalomas***		
Arachnoid cysts	MRI should be repeated only in case of large cysts or localization at risk of hydrocephalus	(24, 46, 47)
Pineal cysts	MRI should be repeated only with cysts >14 mm and/or with an abnormal radiological pattern or clinical symptoms	(45, 48–51)
Choroid plexus cysts	Not require follow-up imaging studies or specific medical management	(52–54)
Vascular anomalies	Neurosurgical consult	(36, 55–57)
Increased hypophyseal volume	Not require follow-up imaging studies or specific medical management	(2, 58, 59)

Laboratory tests include: IGF-1, cortisol, prolactin, FSH and LH, estradiol/testosterone, TSH and FT4.

Overall, there are a few elements that we would like to remark on.

First, all the indications reported in the table refer to brain MRI findings that are not causing any neurological manifestation. In the presence of signs or symptoms suggestive of endocranial hypertension, such as headache, vomit, and arterial hypertension with bradycardia, an MRI evaluation is always warranted, regardless of a history of previous brain findings incidental or not.

Another critical element is that the pituitary gland volume should always be compared to the normal dimensions per age and sex of the patient. In our personal experience, we deal with various inappropriate endocrinological referrals due to misinterpretation of pituitary gland dimensions, especially coming from centers lacking pediatric radiologists. The most typical context is the identification of an enlarged pituitary gland in a teenager who underwent brain MRI because of recurrent headaches but in which no possible underlying elements were found. Therefore, before starting a complete examination of the pituitary gland functionality with a laboratory test and scheduling a follow-up brain MRI, we suggest evaluation of the initial imaging study by a radiologist with expertise in pediatric neuro-imaging.

A third clarification must be made on vascular anomalies. In this case, a neurosurgical consult is necessary because their follow-up and management depend primarily on their correct classification. For example, while cerebral developmental venous anomalies are managed conservatively (56), arteriovenous malformations are usually treated surgically or endoscopically because of their high risk of rupture in the pediatric population (55). A similar approach must also be chosen for asymptomatic Arnold-Chiari type 1 malformations since the timing of their radiological follow-up is currently discussed among specialists, even if the management is known to be conservative (60).

## Conclusions

The purpose of this manuscript is not to underestimate the possible relevance of incidental MRI findings in the diagnostic process of GHD and CPP. Our goal was to highlight that, based on the current literature, most of the follow-up brain MRIs are probably not required in these incidentalomas and that only a few neuroimaging findings are worth subsequent investigations.

## Data Availability Statement

The original contributions presented in the study are included in the article/supplementary material. Further inquiries can be directed to the corresponding author.

## Author Contributions

FB, MM, and FM performed the literature search and wrote the manuscript. EB critically reviewed the manuscript for important intellectual content. GT conceptualized the paper and reviewed and revised the manuscript. All authors approved the final manuscript as submitted and agree to be accountable for all aspects of the work.

## Conflict of Interest

The authors declare that the research was conducted in the absence of any commercial or financial relationships that could be construed as a potential conflict of interest.

## Publisher’s Note

All claims expressed in this article are solely those of the authors and do not necessarily represent those of their affiliated organizations, or those of the publisher, the editors and the reviewers. Any product that may be evaluated in this article, or claim that may be made by its manufacturer, is not guaranteed or endorsed by the publisher.
